# Towards personalized vaccines

**DOI:** 10.3389/fimmu.2024.1436108

**Published:** 2024-10-03

**Authors:** Davide Montin, Veronica Santilli, Alessandra Beni, Giorgio Costagliola, Baldassarre Martire, Maria Felicia Mastrototaro, Giorgio Ottaviano, Caterina Rizzo, Mayla Sgrulletti, Michele Miraglia Del Giudice, Viviana Moschese

**Affiliations:** ^1^ Division of Pediatric Immunology and Rheumatology, “Regina Margherita” Children Hospital, Turin, Italy; ^2^ Research Unit of Clinical Immunology and Vaccinology, Academic Department of Pediatrics (DPUO), IRCCS Bambino Gesù Children’s Hospital, Rome, Italy; ^3^ Department of Clinical and Experimental Medicine, University of Pisa, Pisa, Italy; ^4^ Section of Pediatric Hematology and Oncology, Azienda Ospedaliero Universitaria Pisana, Pisa, Italy; ^5^ Unità Operativa Complessa (UOC) of Pediatrics and Neonatology, “Monsignor A.R. Dimiccoli” Hospital, Barletta, Italy; ^6^ Department of Pediatrics, Fondazione IRCCS San Gerardo Dei Tintori, Monza, Italy; ^7^ Department of Translational Research and New Technologies in Medicine and Surgery, University of Pisa, Pisa, Italy; ^8^ Pediatric Immunopathology and Allergology Unit, Policlinico Tor Vergata, University of Rome Tor Vergata, Rome, Italy; ^9^ PhD Program in Immunology, Molecular Medicine and Applied Biotechnology, University of Rome Tor Vergata, Rome, Italy; ^10^ Department of Woman, Child and of General and Specialized Surgery, University of Campania “Luigi Vanvitelli”, Naples, Italy

**Keywords:** vaccines, vaccinomics and personalized vaccines, system vaccinology, proteomics, genomics, transcriptomics, precision medicine

## Abstract

The emergence of vaccinomics and system vaccinology represents a transformative shift in immunization strategies, advocating for personalized vaccines tailored to individual genetic and immunological profiles. Integrating insights from genomics, transcriptomics, proteomics, and immunology, personalized vaccines offer the promise of enhanced efficacy and safety, revolutionizing the field of vaccinology. However, the development of personalized vaccines presents multifaceted challenges, including technical, ethical, economic, and regulatory considerations. Addressing these challenges is essential to ensure equitable access and safety of personalized vaccination strategies. Despite these hurdles, the potential of personalized vaccines to optimize responses and mitigate disease burden underscores the significance of ongoing research and collaboration in advancing precision medicine in immunization.

## Graphical Abstract

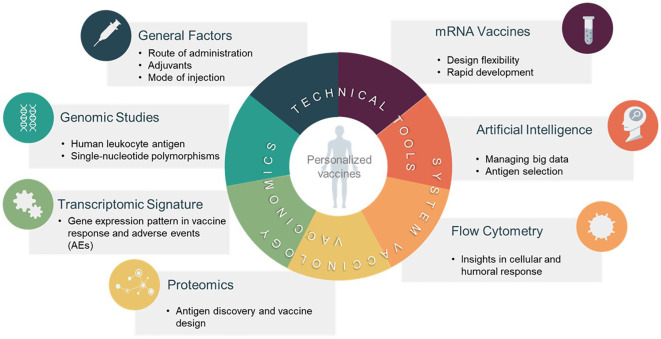

## 1 Introduction

Vaccines are a cornerstone of public health, conferring immunity against various infectious diseases and avoiding millions of deaths annually. However, traditional vaccine approaches have inherent limitations, particularly in addressing individual variability in immune responses and susceptibility to diseases.

Vaccinomics seeks to elucidate the influence of genetic variability on individuals’ immune responses to vaccines. This understanding facilitates the development of personalized vaccination strategies, enabling the customization of vaccines according to individual genetic profiles. Additionally, by identifying genetic markers that predict vaccine efficacy and adverse reactions, and by analyzing how genetic variations among different populations impact vaccine responses and disease susceptibility, it becomes possible to create vaccines that are more effective for specific genetic subgroups. This approach also aims to minimize adverse reactions and enhance the overall efficacy of vaccination programs by taking genetic diversity into account.

Whereas vaccinomics is focused on the interplay between an individual’s genetic makeup and their response to vaccines, system vaccinology investigates the complex interactions and networks within the immune system in response to vaccines. System vaccinology integrates, in a holistic approach, high-throughput technologies (like proteomics, transcriptomics, and metabolomics) to analyze the global changes in the immune system following vaccination and uses computational models to predict vaccine responses and outcomes based on large datasets. Both fields contribute significantly to the development of better, safer, and more effective vaccines, but they do so through different methodologies and perspectives (see **Table 1**). Sharing insights of vaccinomics and system vaccinology, personalized vaccines offer the promise of enhanced efficacy, safety, and coverage, opening a new era of precision medicine in immunization (1).

**Table 1:** Comparison between vaccinomics and system vaccinology.

	Vaccinomics	System Vaccinology
**Scope and Focus**	More focused on the genetic and individual variability in vaccine response. It’s about tailoring vaccines to individuals or groups based on their genetic makeup.	Takes a broader, systems-level view, looking at the overall immune response and the interactions within the immune system. It’s about understanding the global response and mechanisms triggered by vaccines
**Approaches**	Utilizes genomic data, genetic markers, and personalized medicine techniques.	Employs high-throughput technologies, bioinformatics, and computational biology to study immune responses at a systems level.
**End Goals**	Aims for personalized and more effective vaccination strategies tailored to genetic profiles.	Seeks to understand and optimize the complex biological processes and networks involved in vaccine responses for broader and more generalized applications.

This review aims to provide an overview of some aspects of the vaccine personalization process. For the sake of brevity, we have primarily focused on examples related to vaccines against bacterial and viral pathogens; vaccines targeting parasitic and fungal diseases, as well as the expansive field of cancer vaccines, would require a more extensive discussion that falls outside the scope of this review. For further exploration of the topics not covered here, we refer readers to excellent works in the literature that offer a thorough examination of these areas as well (2, 3).

## 2 General factors influencing response to vaccines

Variability in immune response to vaccination is a multifaceted phenomenon influenced by several key factors, including the route of administration, the use of adjuvants, and the mode of injection. The route of vaccination—whether intramuscular, subcutaneous, intradermal, oral, or nasal—plays a crucial role in determining the nature and extent of the immune response. For instance, intramuscular injections are known for inducing robust systemic immunity and are widely used for various vaccines. Subcutaneous injections, on the other hand, often result in slower absorption and prolonged immune activation, while intradermal injections, targeting the dermal layer rich in immune cells, can elicit strong local immune responses. Oral vaccines engage the gut-associated lymphoid tissue (GALT), promoting mucosal immunity, and nasal vaccines stimulate immune responses in the respiratory tract, which is advantageous for respiratory pathogens (4).

Adjuvants, substances incorporated into vaccines to enhance immunogenicity, further modulate the immune response. Common adjuvants like aluminum salts (alum) are known for their ability to boost antibody responses. Oil-in-water emulsions such as MF59 enhance both antibody and cell-mediated immunity. More advanced adjuvants like AS01 and AS03, which are lipid-based, can potentiate both humoral and cellular responses, while synthetic CpG oligodeoxynucleotides mimic bacterial DNA to activate innate immune pathways (5).

The mode of injection also significantly affects the immune response. Traditional needle and syringe methods, while precise, can vary in effectiveness based on administration technique. Jet injectors, which deliver vaccines through high-pressure streams without needles, may enhance immune responses by better targeting the dermal and subcutaneous layers. Additionally, micro-needle patches offer a promising, minimally invasive alternative that targets the skin’s immune-rich layers, potentially boosting both local and systemic immunity (6).

In summary, the interplay between the route of vaccination, adjuvants, and mode of injection is critical in shaping the immune response. Understanding and optimizing these factors is essential for maximizing vaccine efficacy and achieving better protection against infectious diseases.

## 3 The science behind personalized vaccines

Vaccinomics and system vaccinology are medical fields where cutting-edge technologies converge to elucidate the complex interplay between the immune system and vaccine responses. This section aims to provide an overview of some of the most commonly used methods in vaccinomics and system vaccinology, as well as their practical applications in developing personalized vaccines (see **Table 2**). Specifically, we will focus on genomics, transcriptomics, and proteomics studies, along with the use of flow and mass cytometry.

**Table 2:** Table summarizing the main studies mentioned, including the type of vaccine, number of individuals, purpose, and results.

Study	Type of vaccine	Number of individuals	Purpose	Results
**Nishida N, et al. (2018)** (7)	HB vaccine designed based on the HBV genotype C	1193	To identify association between human leukocyte antigen (HLA) alleles and responsiveness to the HB vaccine	Individuals with certain HLA class II alleles (DRB1*01:01, DRB1*08:03, DQB1*05:01, and DPB1*04:02), have shown enhanced humoral immune responses following HB vaccination. Conversely, other HLA alleles, such as HLA-DQB1*04:01, have been associated with reduced vaccine-induced antibody production
**Ovsyannikova IG, et al. (2009) (** 10)	Measles- mumps-rubella (MMR) vaccine containing the attenuated RA27/3 Wistar strain of rubella virus	738	To identify association between specific class I and II HLA markers and haplotypes with rubella vaccine- induced humoral responses	Relationship between the HLA-B27 supertype and lower rubella antibody levels
**Franco LM, et al. (2013)** (13)	Influenza vaccine	247	To identify genes whose genotype affects antibody response	Variation at the level of genes involved in membrane trafficking and antigen processing significantly influences the human response to influenza vaccination
**Dhiman N, et al. (2007) (** 14)	Measles-mumps-rubella vaccine	339	To identify SNP associations in SLAM and CD46 genes with variations in measles antibody response	Heterozygous rs2724384 polymorphism in CD46 gene is associated with a two-fold decrease in measles vaccine-induced antibody levels
**Dhiman N, et al. (2008) (** 15)	Measles-mumps-rubella vaccine	190	To identify associations between SNPs in toll-like receptors and immune responses to measles vaccine	Heterozygous variants for rs3775291 and rs5743305 of the TLR3 gene were associated with low antibody and lymphoproliferative responses to measles vaccination
**Ovsyannikova IG, et al. (2014)** (17)	Iinactivated influenza vaccine	159	To examine correlations between leptin concentrations, influenza vaccine-induced immune responses and an immunosenescence marker (TREC), and to examine associations between leptin-related gene polymorphisms and vaccine-induced immunity in older individuals.	Several SNPs in the leptin and leptin related genes are associated with variations in influenza-specific HAI and B-cell responses
**Chan CY, et al. (2017)** (19)	Yellow fever live-attenuated vaccine	68	To study the molecular correlates of reactogenicity or adverse events	Early activation of innate immune genes at day 1, but not at day 3 after vaccination, was directly correlated with adverse events
**Querec TD, et al. (2009)** (20)	Yellow fever vaccine	15	To identify early gene ‘signatures’ that predicted immune responses using a systems biology approach	Complement protein C1qB and eukaryotic translation initiation factor 2 alpha kinase 4 (EIF2AK4) showed a strong correlation with YF-17D CD8(+) T cell responses; B cell growth factor TNFRS17 predicted the neutralizing antibody response
**Gómez-Carballa A, et al. (2021)** (22)	Live attenuated pentavalent human-bovine reassorted vaccine RotaTeq^®^ (RV5)	32	To compare the transcriptome of children affected by community-acquired RV infection, children immunized with RV5 and healthy controls	Transcriptomic profiling has revealed differential expression of genes between RV5 recipients and control subjects, some of which are associated with intussusception and midgut abnormalities
**Price JV, et al. (2013) (** 25 **)**	Influenza vaccine	76	Characterization of influenza vaccine immunogenicity using influenza antigen microarrays	An inverse correlation between several peptide epitopes and age and neutralization titers was demonstrated
**Furman D, et al. (2013) (** 26 **)**	Influenza vaccine	89	To identify markers that predict influenza vaccine responsiveness	Using machine learning, nine variables that predict the antibody response were identified
**Davies DH, et al. (2007) (** 24 **)**	Smallpox vaccine (Dryvax^®^)	25 individuals vaccinated, 5 archival smallpox sera	To derive a proteome-wide view of the antibody response of humans to Dryvax^®^ and variola using protein microarrays	The pattern of antigens recognized by sera from convalescent and vaccinated individuals exhibited remarkable similarities; a significant component of the antibody response is not involved in virus neutralization, although these antigens should be considered alongside the envelope proteins as potential candidates for diagnostic and vaccine applications.

### 3.1 Genomic insights into vaccine efficacy

#### 3.1.1 HLA alleles and vaccine response

The human leukocyte antigen (HLA) system plays a pivotal role in modulating immune responses to vaccines. HLA molecules, encoded by a highly polymorphic gene complex located on chromosome 6, are integral to antigen presentation and recognition by T cells. Genetic variants within the HLA loci affect vaccine-induced immune responses, including antibody production, T cell activation and cytokine secretion. Understanding the relationship between HLA polymorphisms and vaccine efficacy is essential for optimizing vaccination strategies and improving individualized immunization outcomes.

Approximately 5-10% of individuals who are vaccinated with a hepatitis B (HB) vaccine designed based on the hepatitis B virus (HBV) genotype C fail to acquire protective levels of antibodies. Studies have revealed a significant association between human leukocyte antigen (HLA) alleles and responsiveness to the HB vaccine. For instance, individuals with certain HLA class II alleles (DRB1^*^01:01, DRB1^*^08:03, DQB1^*^05:01, and DPB1^*^04:02), have shown enhanced humoral immune responses following HB vaccination. Conversely, other HLA alleles, such as HLA-DQB1^*^04:01, have been associated with reduced vaccine-induced antibody production (7).

Regarding smallpox vaccine, a population-based study suggested that the HLA-B*1501 allele (p = 0.016) was highly correlated with vaccinia virus-specific cellular IFN-γ Elispot T cell responses after smallpox vaccination. By applying this HLA-based approach, it is possible to identify specific epitopes in vaccinia virus, that are restricted for class I B*1501 (B15 supertype) (8). These peptides can be assessed as new smallpox vaccines while having the benefits of immunogenicity and effectiveness, reduced frequency of side effects, and the ability to produce them at low cost (9).

Moreover, two studies show a relationship between the HLA-B7 supertype and higher measles vaccine-induced antibody levels, and a relationship between the HLA-B27 supertype and lower rubella antibody levels (10, 11). As above, personalized selection of viral peptides presented by B7 supertype alleles could be a promising strategy for novel and more effective MMR vaccines.

#### 3.1.2 Genetic variants and vaccine effectiveness

Beyond the HLA system, a myriad of genes spanning diverse biological pathways are implicated in modulating vaccine responses. These genes encompass a spectrum of functional categories, including but not limited to, immune-related genes, metabolic enzymes, signaling molecules, and cellular receptors. Polymorphisms within these non-HLA genes have emerged as significant determinants of vaccine-induced immune responses, exerting influence on antigen processing and presentation, innate immune activation, and adaptive immune priming.

Genome-wide association studies (GWAS) have identified several single-nucleotide polymorphisms (SNPs) associated with variability in influenza vaccine effectiveness. For example, polymorphisms in genes encoding components of the innate immune system, such as toll-like receptors (TLRs) and cytokines, have been implicated in modulating influenza vaccine responsiveness. Poland et al. found several SNPs in coding and non-coding regions of cytokines and cytokine receptors associated with antibodies production after vaccination (12). More interestingly, Franco et al. combined genotype, gene expression and antibody titer information to identify genes whose genotype affects antibody response. They identified 20 genes, most of them not specifically linked to the immune system, but rather to intracellular transport and membrane trafficking (NAPSA, GLMP, GM2A, DYNL1, SNX29, TAP2, FGD2) (13). The impact of SNPs on the vaccine induced humoral immune response presents two main concerns: the first one is that the replication of GWAS results is often difficult to achieve, while the second concern is that GWAS demonstrates statistical but not causal associations. However, these findings demonstrate the complex interplay between host genetics and vaccine-induced immune responses, highlighting the innovative potential of personalized vaccination strategies based on individual genetic profiles.

SNPs investigations can also be conducted with a targeted approach. For example, measles infection requires interaction with cellular receptor CD46; SNPs in CD46 genes might therefore influence the immune response to measles vaccine. Actually, heterozygous rs2724384 polymorphism in CD46 gene is associated with a two-fold decrease in measles vaccine-induced antibody levels (14). Toll-like receptors (TLRs) represent the critical “bridge” between innate and adaptive immunity to viral pathogens and they are mainly responsible for the initiation of the immune response. In agreement with this observation, heterozygous variants rs3775291 and rs5743305 of the TLR3 gene are associated with low antibody and lymphoproliferative responses to measles vaccination. In addition, SNPs in MyD88, an intracellular molecule that associates with TLRs, also show an association with antibody levels and IL-10 (15).

Among genes not directly linked to the immune system, it is worth mentioning the role of leptin indeed. Leptin is a hormone released by the adipose tissue, whose primary role is likely to regulate long-term energy balance. Chronically elevated leptin levels are associated with obesity and inflammation-related diseases including metabolic syndrome and cardiovascular diseases. Obesity and metabolic syndrome have a substantial impact on immunity and pathogen defense and are associated with an overall negative impact on vaccine efficacy (16). It can therefore be hypothesized that quantitative or qualitative alterations in leptin production may affect susceptibility to infections and poor vaccine outcomes. In individuals immunized with inactivated influenza vaccine that contained A/California/7/2009/H1N1 virus, while leptin concentration has no association with hemagglutination antibody inhibition (HAI), several SNPs in the leptin and leptin related genes are associated with variations in influenza-specific HAI and B-cell responses (17).

### 3.2 Transcriptomic signatures of vaccine responsiveness

Transcriptomics encompasses the study of all RNA transcripts produced by the genome within a cell or tissue at a given moment. This discipline offers insights into the dynamic regulation of gene expression underlying cellular processes, developmental pathways, and disease states. By employing high-throughput sequencing technologies such as RNA sequencing (RNA-seq), transcriptomics enables comprehensive characterization of the transcriptome, including messenger RNA (mRNA), non-coding RNA (ncRNA), and alternatively spliced isoforms.

#### 3.2.1 mRNA expression profiles and yellow fever vaccine response

The yellow fever vaccine (YF-17D) is one of the most effective vaccines available. A single injection of YF-17D induces an incredibly wide array of immune responses, including cytotoxic T lymphocytes (CTLs), helper T lymphocytes, and neutralizing antibodies that can persist for up to 30 years (18). Nevertheless, despite its efficacy, little is known about how this vaccine induces effective immune responses.

Transcriptomics has demonstrated that an unexpected activation of molecular events, usually associated with an anti-viral response, occurs after YF-17D vaccination. In particular, molecules involved in innate sensing of viruses, such as TLR7, cytoplasmic receptors of 2,5′-OAS family, RIG-I, and MDA-5, as well as transcription factors that regulate type I interferons (*IRF7, STAT1*), were induced, usually at day 3 after vaccination. Transcription of such genes has dubious correlation with the magnitude of immune response, but it is apparently associated with emergence of adverse events (AEs). About 25% of vaccinated people may experience mild symptoms such as moderate fever, headache, muscle pain that appear 5-10 days after vaccination. Rarely, approximately 1 time every 150,000 doses, the vaccine can cause serious reactions such as meningoencephalitis, encephalomyelitis and Guillain Barré syndrome. In a study of transcriptomics conducted on 68 patients, early activation of innate immune genes at day 1, but not at day 3 after vaccination, was directly correlated with AEs (19). The use of genomic profiling thus provides molecular insights into the biology of AEs that might potentially leads to the development of safer vaccines.

Investigations into the immunogenicity of the YF-17D vaccine revealed the identification of a specific genetic profile. This profile included the presence of complement protein C1qB and eukaryotic translation initiation factor 2 alpha kinase 4 (EIF2AK4), which play a key role in orchestrating the integrated stress response. Remarkably, this genetic signature showed a strong correlation with YF-17D CD8(+) T cell responses and acted as a predictive indicator too, achieving an accuracy of up to 90%. Additionally, a distinct genetic signature, featuring B cell growth factor TNFRS17, accurately predicted the neutralizing antibody response, achieving a remarkable accuracy rate of up to 100%. These findings underscore the invaluable contribution of systems biology methodologies in forecasting vaccine efficacy (20).

#### 3.2.2 Gene expression patterns and influenza vaccine response

Recent investigations have highlighted gene expression patterns linked to influenza vaccine. Disparities have been observed in the immune profiles of individuals receiving inactivated influenza vaccine (IIV) in comparison to those receiving live attenuated influenza vaccine (LAIV). IIV administration has generally been associated with heightened transcriptional activity of B cells, plasmablasts, plasma cells, and conventional dendritic cell (DC) populations. Additionally, early transcriptional signatures observed in IIV recipients, encompassing interferon signaling, antigen processing, presentation, and IL-6 regulation, have been predictive of hemagglutination inhibition (HAI) production post-vaccination. Furthermore, the incorporation of adjuvants into IIV formulations may elicit distinct signature patterns specific to each adjuvant, alongside the antigen. Notably, adjuvant AS03 has been shown to induce proliferation activity in natural killer (NK) cells and modulate interferon signaling, antigen processing, and presentation several days post-vaccination. Nevertheless, the relationship between adjuvant signatures and vaccine-conferred protection warrants further elucidation.

Conversely, the transcriptional signature associated with LAIV administration is characterized by heightened activity of plasmacytoid DCs, T cells, and NK cells modules; of note, interferon-signaling pathways are induced seven days post-vaccination. Differential transcriptional signatures between LAIV and IIV recipients have revealed the upregulation of five genes representing an interferon-stimulated gene response. Intriguingly, LAIV shows a transcriptional signature more similar to the yellow fever vaccine one, that is another live attenuated virus vaccine, rather to the signature observed with IIV.

Additionally, transcriptional signatures post-influenza vaccine exhibit variations across different demographic groups, including children, young adults, older adults, racial demographics, and between genders. Further investigations are warranted to comprehend the fundamental differences in immune responses among vulnerable populations (21).

#### 3.2.3 mRNA profiling and risk of AEs with Rotavirus vaccine

Europe has licensed two distinct vaccines for immunization against Rotavirus (RV): the live attenuated pentavalent human-bovine reassorted vaccine RotaTeq (RV5) and the live attenuated human vaccine Rotarix (RV1). RV5 consists of a combination of five human/bovine reassorted RV strains with limited replication in the gut, while RV1 is derived from a single human live attenuated strain with robust gut replication capabilities. In light of historical associations between earlier RV vaccines and intussusception, large-scale safety studies were conducted on RV5 and RV1 prior to approval. However, the relationship between RV vaccination and intussusception remains ambiguous, with several studies failing to demonstrate an increased incidence of intussusception following RV5 administration. Consensus now suggests that the benefits of RV vaccination far outweigh the minimal risk of intussusception. Transcriptomic profiling has revealed differential expression of genes between RV5 recipients and control subjects, some of which are associated with intussusception and midgut abnormalities. For instance, the upregulation of the APC gene post-vaccination has been linked to the development of jejunal adenoma-induced intussusception. This gene expression signature may help elucidate the reported higher risk of intussusception in vaccinated children. Future vaccine development efforts could target these genes to enhance vaccine safety, particularly for children experiencing intussusception post-vaccination (22).

### 3.3 Proteomics for antigen discovery and vaccine design

Proteomics is concerned with the comprehensive study of proteins expressed by a biological system at a given moment. It encompasses the identification, quantification, and characterization of proteins, including their post-translational modifications and interactions, in order to clarify their roles in physiological processes, disease mechanisms, and therapeutic responses. Proteomic techniques, such as mass spectrometry-based protein profiling and protein-protein interaction analysis, enable the systematic investigation of complex protein networks within cells, tissues, and biological fluids.

#### 3.3.1 Proteomic approach to respiratory syncytial virus vaccine design

RSV type A and type B are a leading cause of respiratory illness in infants, young children, and the elderly, yet efforts to develop an effective vaccine have been hindered by challenges in identifying suitable antigens; despite these efforts, the first recombinant-adjuvanted RSV vaccine in adults aged 60 years and older was authorized only few months ago, in May 2023. A second RSV vaccine was approved in late 2023, targeting not only adults over 60 but also pregnant women between the 32nd and 36th week of gestation, providing protection to future newborns up to 6 months of age. This vaccine is a non-adjuvanted bivalent RSV type A and B vaccine composed of two preF proteins (RSVpreF) to be administered as a single-dose injection.

Recently, proteomics combined with immunoinformatics was used to draw a polyvalent vaccine capable of inducing a substantial immune response against both RSV-A and RSV-B types (23). Four highly virulent proteins have been identified: phosphoprotein (P protein), nucleoprotein (N protein), fusion glycoprotein (F protein), and major surface glycoprotein (mG protein). These proteins were analyzed to isolate potential epitopes capable of stimulating an immune response, characterized by high antigenicity, non-allergenicity, non-toxicity, conservancy, and human proteome non-homology. The epitopes were filtered on the basis of their ability to interact with the most widespread MHC class I and II alleles. The vaccine sequence was constructed by linking the most promising epitopes with appropriate linkers. Additionally, an adjuvant sequence was conjugated in the appropriate position. Mechanistic approaches to imitate and predict the potential immune response generated by the administration of vaccine were determined through immune simulations. Although this is a pre-clinical study that needs further *in vitro* and *in vivo* experiments to validate its efficacy, it represents one of the most promising approaches for the creation of next-generation RSV vaccines with improved efficacy and safety profiles.

#### 3.3.2 Analyzing the human proteomic response to vaccine

In recent years, there has been a notable surge in the utilization of proteomic methodologies to investigate vaccine immune responses. Initial efforts in proteomic analysis mirrored the methodologies employed in RNA studies, primarily by protein microarrays. For instance, the response to vaccinia virus vaccine was explored by microarrays encompassing the entire proteome of vaccinia. This approach aimed to probe antibody reactivity following primary infection or smallpox vaccine boosting, as well as in convalescent patients’ smallpox sera. By indirect immunofluorescence, antibodies targeting specific epitopes present on the microarray were delineated, facilitating both quantitative and qualitative assessments of the antibody response. A significant proportion of the recognized antigens comprised core proteins (such as A10, L4, and I1), proteins implicated in intracellular morphogenesis (e.g., A11, D13), and the A-type inclusion protein, WR148. Notably, the pattern of antigens recognized by sera from convalescent and vaccinated individuals exhibited remarkable similarities. These findings suggest that a substantial component of the antibody response may not contribute to virus neutralization. Nonetheless, these antigens warrant consideration alongside envelope proteins as potential candidates for diagnostic and vaccine development applications (24).

A similar methodology was employed to investigate the serological response following influenza vaccine. By comparing sera obtained from both young and elderly individuals immediately before and one month after vaccination, peptide-array reactivity showed a significant correlation with age and neutralization titers. Notably, it was demonstrated an inverse correlation between several peptide epitopes and age and neutralization titers, indicating a potential association between reactivity to these epitopes and age-related deficiencies in response to H1N1 influenza (25).

Peptide arrays offer a versatile platform to identify specific biomarkers prior to vaccination that may serve as predictors of vaccine response. Analysis of sera collected from patients prior to influenza vaccine revealed several hemagglutinin-linear epitope specificities that show a robust negative correlation with the hemagglutination inhibition (HAI) response to influenza vaccination. Moreover, these antibody specificities are frequently associated with age, potentially contributing to the overall diminished response observed in aged individuals. This phenomenon is likely attributed to previous exposure to influenza virus in elderly individuals compared to younger subjects. Leveraging this system enables rapid discrimination between good and poor responders based on their repertoire of pre-vaccination antibodies targeting HA protein regions. Consequently, this represents a promising approach for customizing vaccine delivery strategies across different age groups or during pandemics (26).

In the context of tetanus vaccination, high-resolution liquid chromatography tandem mass spectrometry (LC-MS/MS) proteomic analyses of serum antibodies were utilized to characterize the human serum IgG repertoire following booster vaccination. Among the numerous specific clonotypes that expanded shortly after booster administration (on day 7), only three clonotypes remained detectable after 9 months, collectively accounting for over 40% of the antibody response. Notably, it was observed that the post-booster antibody repertoire largely resembled the pre-vaccination serum repertoire. Clonotypes of IgG with higher affinity to vaccine antigens were found to bind the same linear epitope on tetanus toxoid, thereby occluding the binding site utilized by the toxin for cell entry. This observation provides a potential mechanistic explanation for the protective efficacy conferred by the tetanus vaccine (27).

### 3.4 Cytometry to predict vaccination outcomes

Flow cytometry plays a key role in vaccine personalization by enabling detailed analysis of immune cell populations and their responses to antigens. The precise identification of cellular phenotypes and functions, including the identification of specific subsets of T cells, B cells and antigen-presenting cells critical for mounting an effective immune response, provides insight into the variability of immune responses between individuals. Indeed, several studies suggest that phenotypic characterization of the T and B cell provides a better definition of correlates of vaccine protection than serological correlates alone (28). For instance, polyfunctional T-cells, characterized by the simultaneous expression of multiple functional markers such as IFN-γ, IL-2, TNF, MIP-1β, and CD107a have been correlated with protective immunity in vaccinations such as Leishmania or Yellow Fever (29). Other studies investigate B cells subsets perturbation in children with perinatally HIV infection, showing as the expansion of double-negative and activated memory B cells correlates with a reduced response to meningococcal B vaccination (30). Finally, Amodio et al. analyzed the humoral and cellular response following the BNT162b2 mRNA COVID-19 vaccine in a cohort of patients affected by inborn errors of immunity, showing as four patients, despite a good post-vaccine seroconversion, did not develop any specific CD4+CD40L+ T cellular response, suggesting that the evaluation of vaccine-induced immunity in this population should also include quantification of Ag-specific T cells (31).

Mass cytometry, also known as cytometry by Time-Of-Flight (CyTOF), represents an advanced flow cytometry platform that employs elemental mass spectrometry to detect metal-labeled antibodies bound to intracellular or extracellular antigens on individual cells. A fundamental distinction between flow and mass cytometry is that, in mass cytometry, fluorophores are replaced with isotopically pure heavy metal atoms, which are typically absent from biological systems. The primary advantage of CyTOF lies in its capacity to analyze a greater number of parameters per panel compared to conventional cytometry techniques. This capability enhances the understanding of complex and heterogeneous cell populations without the necessity for numerous intricate and overlapping panels. Recently, CyTOF has been utilized to explore the humoral response to SARS-CoV-2 mRNA vaccines by examining the memory B cell population. Circulating memory B cells in the blood can be categorized into CD45RB+ and CD45RBlow memory cell subsets, with stable differences in CD45RB sialylation enabling the tracking of activated B cells and plasmablasts derived from these subsets. Notably, the majority of circulating memory B cells that recognize SARS-CoV-2 following infection or mRNA vaccination are predominantly found within distinct populations of CD23+CD45RBlow and atypical CD11c+CD45RBlow B cells. These findings indicate that multiple subsets of CD45RBlow non-classical B cells play a crucial role in the immune response to both COVID-19 and mRNA vaccination (32).

Even more specifically, single-cell RNA sequencing (scRNA-seq) allows the comprehensive analysis of gene expression at the individual cell level and the identification and characterization of heterogeneous cell populations within the immune system, providing insights into the specific cellular responses to vaccination, thus playing a crucial role in the personalization of vaccines. By analyzing the transcriptomes of single cells, scRNA-seq can reveal the presence of rare immune cell subsets, identify novel biomarkers, and uncover mechanisms of immune activation and regulation that are not discernible through bulk RNA sequencing. This detailed understanding of the immune landscape facilitates the design of tailored vaccines that can elicit robust and specific immune responses, optimizing efficacy and safety for individual patients particularly those with special vaccination needs (33, 34).

## 4 mRNA vaccines in the context of personalized vaccination

mRNA vaccines stimulate immune responses by delivering synthetic mRNA, which encodes antigens of the pathogen, into antigen-presenting cells. These antigens are then processed and presented on the cell surface or secreted in interstitial space, where they are recognized by immune cells, triggering protective humoral and cytotoxic T-cell responses. A wealth of reviews and research articles have been published detailing preclinical development and clinical outcomes of coronavirus vaccines and related products (35).

mRNA vaccines have emerged as a promising tool for personalized vaccination, offering unique advantages in both design flexibility and rapid development. This technology allows for the rapid generation of vaccines tailored to target various infectious diseases, including emerging pathogens or those with high mutation rates such as influenza or coronaviruses. Adaptability is indeed a unique advantage of mRNA vaccines, enabling swift modifications to address new variants or strains of pathogens. This adaptability is particularly crucial in the context of personalized vaccination, where individual genetic variations or immunological profiles may necessitate customized vaccine formulations, potentially enhancing vaccine efficacy and safety while minimizing adverse reactions. Moreover, mRNA vaccines offer a safer alternative to conventional vaccine approaches, as they do not require the use of live pathogens or adjuvants, reducing the risk of vaccine-associated complications in specific categories, such as immunocompromised individuals, elderly patients, pregnant women, and pediatric populations.

## 5 Harnessing technology for personalized vaccines

The promise of personalized vaccines announces a shift in the prevailing paradigm of vaccinology, where worldwide immunization practices provide the administration of identical vaccine formulations, doses, and schedules across entire populations, barring contraindications. This conventional approach operates on the assumption of uniform immune responses, comparable levels of immunity — either antibody-mediated or cell-mediated — and equivalent antigenic requirements for immunity development, as well as uniform risk profiles for all individuals. Conversely, the advent of personalized vaccines aims to optimize immunogenicity while minimizing vaccine failures or adverse reactions, particularly in individuals predisposed to severe disease or complications.

While this paradigm shift holds a revolutionary potential, it also brings numerous technical, ethical, economic, and privacy concerns regarding the collection, storage, and use of personal data.

### 5.1 Managing big data

The integration of OMICs data, encompassing genomics, transcriptomics, proteomics, and mass cytometry, is transforming the landscape of personalized vaccine development by providing a granular and comprehensive understanding of individual biological profiles (36). High-throughput assays produce extensive datasets that necessitate the use of advanced computational and mathematical tools for thorough analysis. This is particularly crucial when dealing with deep sequencing data. With the ever-growing number of analytical tools available, the main challenge lies not in collecting data but in analyzing it effectively. To fully leverage these technologies, there is a critical need for specialized training programs for biologists and immunologists. Traditional training in these fields often does not include the necessary skills in data science, bioinformatics, and systems biology. Thus, interdisciplinary training programs that cover computational techniques, statistical analysis, and the practical application of bioinformatics tools are crucial. These programs should also emphasize the principles of big data management and the ethical considerations of handling large-scale biological data (37, 38). Currently, some research groups focus on creating computational infrastructures and tools for immunology analysis. For instance, the National Institute of Allergy and Infectious Diseases (NIAID) of the US National Institutes of Health (NIH) has established the Human Immunology Project Consortium to assemble large datasets on human subjects in various conditions (HIPC; http://www.humanprofiling.org). Moreover, the complex nature of the immune response and the various ways it can be analyzed in modern immunology necessitate a standardized approach for knowledge sharing among scientists worldwide. This standardization is crucial for advancing our understanding of immunity.

### 5.2 Artificial intelligence for personalized vaccines

Artificial intelligence (AI) has emerged as a key player in modern healthcare. In recent years, in fact, its uses have been increasingly represented, from diagnostic tools to predictive analytics and personalized medicine. The pharmaceutical industry is widely benefiting from AI for the development of new drugs and vaccines candidates. Traditionally, vaccine development it is a long and complex process that can take 10 to 15 years. This involves several stages, from pre-clinic research to clinical trials and regulatory approval. AI has the potential to significantly reduce these timelines. Indeed, AI can analyze vast datasets, including genomic and proteomic information and chemical properties, to predict how different compounds might behave and interact. This can help the initial phases of drug discovery, allowing pharmaceutical companies to allocate resources more efficiently and increasing the likelihood of bringing impactful drugs to market (39, 40). In particular, speaking of vaccines, machine learning (ML) can assist several stages of vaccine design, supporting a fast and accurate target selection during the initial phase. Here ML algorithms serve for the identification and optimization of B and T cell epitopes and can improve the study of correlates of protection by helping assess quality and specificity of vaccine-induced cellular and humoral responses (41). Furthermore, one of the most promising developments is that AI aids in personalized medicine by analyzing patient data to identify specific genetic markers and biomarkers that can influence drug response. This allows for the development of targeted therapies tailored to individual patients. For instance, different strains of a virus may circulate in different regions of the world, and AI can help design vaccines that are more effective against the predominant strains in a given geographical area. This personalized medicine approach not only enhances the effectiveness of treatments but also minimizes adverse effects, reducing the overall environmental impact associated with the production and disposal of pharmaceuticals. In addition, AI could also monitor the efficacy of vaccines over time, improving post marketing surveillance (42). In essence, AI in target identification brings a level of precision and efficiency that has the potential to revolutionize the drug discovery process and build the way for more effective and personalized therapeutic and prevention interventions (43).

### 5.3 Ethical considerations

Ensuring equitable access to personalized vaccines is crucial, particularly in resource-limited settings. The customized nature of these vaccines may imply higher manufacturing costs and logistical challenges compared to traditional vaccines, thus necessitating strategic measures to maximize their public health impact and mitigating potential disparities in healthcare delivery. Regulatory rules involve significant challenges to the widespread adoption of personalized vaccines. The approval process of a vaccine is complex and time-consuming, demanding robust evidence of safety, efficacy, and quality control measures. Streamlining regulatory pathways and the establishment of clear development guidelines are essential to accelerate the translation of personalized vaccines into clinical practice. Finally, the protection of patient confidentiality and data privacy and security are critical issues in personalized vaccine development. Given the reliance on sensitive patient data collection and analysis, the implementation of robust data governance frameworks and compliance with privacy regulations are imperative to maintain trust and the safeguard of patient information.

## 6 Future directions and concluding remarks

The advancement of personalized vaccines marks a pivotal transition in the landscape of vaccinology, offering the prospect of personalized vaccines tailored to individual genetic and immunological profiles. This paradigm shift holds a great promise for optimal vaccine efficacy and safety, prospecting a new era of precision medicine in immunization. Thanks to growing knowledge in genomics, transcriptomics, proteomics, and immunology fields, personalized vaccines aim to maximize immunogenicity while minimizing adverse reactions, particularly in individuals susceptible to severe disease or complications.

However, the development of personalized vaccines is accompanied by heterogeneous challenges from ethical to economic and regulatory domains. requiring proper solutions to ensure equitable access, affordability, and safety of personalized vaccines. Strategies for streamlining regulatory pathways, reducing manufacturing costs, and enhancing data privacy and security are imperative to facilitate the widespread adoption of personalized vaccination strategies.

Despite these challenges, the potential benefits of personalized vaccines in public health are undeniable. Personalized vaccines can overcome the limitations of traditional vaccine approaches, starting a new era with more effective, targeted, and individualized immunization strategies. As we navigate the complexities of personalized vaccine development, collaboration across interdisciplinary fields and concerted efforts in research, policy, and practice will be essential for the safeguard of global health.
